# Genetic variants in lipid metabolism are independently associated with multiple features of the metabolic syndrome

**DOI:** 10.1186/1476-511X-10-118

**Published:** 2011-07-18

**Authors:** Cécile M Povel, Jolanda MA Boer, Sandra Imholz, Martijn ET Dollé, Edith JM Feskens

**Affiliations:** 1 Centre for Nutrition and Health (CVG), National Institute for Public Health and the Environment (RIVM), Bilthoven, the Netherlands; 2 Division of Human Nutrition, Wageningen University, Wageningen, the Netherlands; 3 Laboratory for Health Protection Research (GBO), National Institute for Public Health and the Environment (RIVM), Bilthoven, the Netherlands

**Keywords:** HDL-cholesterol, abdominal obesity, metabolic syndrome, CETP, APOE

## Abstract

**Background:**

Our objective was to find single nucleotide polymorphisms (SNPs), within transcriptional pathways of glucose and lipid metabolism, which are related to multiple features of the metabolic syndrome (MetS).

**Methods:**

373 SNPs were measured in 3575 subjects of the Doetinchem cohort. Prevalence of MetS features, i.e. hyperglycemia, abdominal obesity, decreased HDL-cholesterol levels and hypertension, were measured twice in 6 years. Associations between the SNPs and the individual MetS features were analyzed by log-linear models. For SNPs related to multiple MetS features (P < 0.01), we investigated whether these associations were independent of each other.

**Results:**

Two SNPs, *CETP Ile405Val *and *APOE Cys112Arg*, were associated with both the prevalence of low HDL-cholesterol level (*Ile405Val *P = < .0001; *Cys112Arg *P = 0.001) and with the prevalence of abdominal obesity (*Ile405Val *P = 0.007; *Cys112Arg *P = 0.007). For both SNPs, the association with HDL-cholesterol was partly independent of the association with abdominal obesity and vice versa.

**Conclusion:**

Two SNPs, mainly known for their role in lipid metabolism, were associated with two MetS features i.e., low HDL-cholesterol concentration, as well as, independent of this association, abdominal obesity. These SNPs may help to explain why low HDL-cholesterol levels and abdominal obesity frequently co-occur.

## Introduction

The metabolic syndrome (MetS) is a common multi-component condition including abdominal obesity, dyslipidemia, hypertension, and hyperglycemia. It is associated with an increased risk of cardiovascular disease and type 2 diabetes [1]. A central question in understanding the MetS is why these traits cluster together [1]. The clustering may be explained by a complex physiological cascade of events, in which the occurrence of one trait initiates the occurrence of a second. Alternatively, a causative factor common to several metabolic traits may explain the clustering. This factor could be either of genetic or environmental nature [2].

Family and twin studies indicate that the different features of the MetS share a common genetic component [2-5]. Twin studies show that the correlation between the features of the metabolic syndrome is higher in monozygotic compared to dizygotic twins [2,6,7]. Family studies also show significant genetic correlations between the different features of the metabolic syndrome [4]. Heritability estimates of the MetS itself range from 13-27% [3-5]. However, despite the evidence from these heritability studies, only a few single nucleotide polymorphisms (SNPs) have been linked to multiple features of the MetS[1].

Disturbances in lipid and glucose metabolism may lead to the development of one or more MetS features [8]. Therefore, genes involved in these pathways are potentially pleiotropic for multiple MetS features. In a population based cohort study, we studied 373 SNPs mainly selected from transcriptional pathways of glucose and lipid metabolism, and their association with multiple features of the MetS.

## Methods

### Study population

The Doetinchem Study is a population-based cohort study on lifestyle, biological risk factors and chronic diseases [9]. Between 1987 and 1991, 12404 subjects, aged 20-59, all inhabitants of Doetinchem, a town in a rural area in east of the Netherlands, were enrolled in the baseline cohort. A random sub-sample of this cohort (63%) was invited for a second measurement round (1993-1997; response 79%) and for a third measurement round (1998-2002; response 75%). Overall, the Doetinchem Cohort comprises 4662 persons with repeated measurements.

Pregnancy and alteration in smoking behavior are factors that influence body weight and therewith the MetS. Therefore, subjects of the Doetinchem Cohort who changed their smoking habits (n = 750), who had missing data on smoking status (n = 11) or who where pregnant at the time of measurement (n = 122) were excluded from the current study. This resulted in a final study population of 3779 subjects. The second and third measurement rounds included glucose and waist circumference measurements and were used for the present study. All participants gave written informed consent and approval was obtained from local Medical-Ethical Committees.

### Measurements

During each measurement round, a questionnaire on lifestyle factors was administered and anthropometric and biochemical variables were measured. For a more detailed description see [10].

During the second and third visit waist circumference was measured according to written instructions based on WHO criteria for waist measurement (1989). Waist circumference was determined to the nearest 0.5 cm, at midway between the lowest rib and the iliac crest, with subjects in standing position and after breathing out gently. Waist circumference was measured in duplicate and the mean of the two measurements was taken. Blood pressure (BP) was measured in each round, with the subject in sitting position using a random-zero sphygmomanometer. Systolic pressure was recorded at the appearance of sounds (first-phase Korotkoff) and diastolic blood pressure was recorded at the disappearance of sounds (fifth-phase Korotkoff). BP measurement was repeated and values were averaged. During the physical examination, regular audits were performed to check adherence to the BP measuring protocol (e.g. resting time, adequate cuff size).

Non-fasting blood samples were taken by venapuncture for all subjects. Blood samples were fractionated into serum, buffy coat and erythrocytes and subsequently stored at -30°C until further use. Plasma glucose levels were measured as described by Tietz [11]. HDL-cholesterol was measured in EDTA-plasma until 1998, and from 1998 onwards in serum, at the Lipid Reference Laboratory (LRL) of the university Hospital Dijkzigt in Rotterdam, using standardized enzymatic methods. Performance for enzymatic HDL-cholesterol measurements fulfilled National Cholesterol Education Program (NCEP) recommendations throughout the entire study period.

Genomic DNA was extracted from the buffy coat fraction with a salting out method. A total of 139 subjects were not eligible for genotyping, mainly because of failure to extract DNA or unavailability of buffy coats. For 3640 subjects, 401 SNP across 270 candidate genes were genotyped. A set of 383 SNP's across 253 candidate genes, passed the Illumina design tool and were genotyped with the Illumina Golden Gate assay using the Sentrix Array Matrix platform (Illumina Inc, San Diego, California) [11]. 18 Additional SNPs were genotyped by KBioscience (Hoddesdon, Hertfordshire, UK) using the KASPar chemistry, which is a competitive allele specific PCR SNP genotyping system using FRET quencher cassette oligonucleotides http://www.kbioscience.co.uk). Two SNPs (rs7412 and rs429358 in APOE) that failed in the Illumina Golden Gate assay were successfully re-genotyped with Taqman assay.

A detailed description of the SNP selection procedure and a full SNP list have been published elsewhere [10]. In short, 270 candidate genes were selected by a pathway-driven approach, with emphasis on regulatory pathways that control fatty acid, glucose, cholesterol and bile salt homeostasis [10]. The selection procedure started from the master regulator genes encoding nuclear receptors (PPARs, LXR, NR1H4) and transcription factors (SREBPs) and continued by selecting their co-activators, co-repressors and target genes. In addition, hormonal receptors (insulin receptor), their down-stream signaling proteins and genes involved in β-signaling were selected. For each gene out of these pathways, 1-7 SNPs most likely to carry functional properties were selected. For 26 SNPs genotyping was unsuccessful. In addition, 33 SNPs were not in Hardy Weinberg Equilibrium (HWE). Verification was carried out in a random sample (n = 96) for the eight SNPs (24%) that deviated most strongly from HWE. All yielded the same results, except for 2 SNPs, which were therefore excluded [10]. After the exclusion of subjects with genotype failure or discordance on gender control (n = 65), 3575 subjects were available for data analyses. Finally, data on 373 SNPs in 254 genes were available for 3575 subjects.

### Statistical analyses

Abdominal obesity, low HDL-cholesterol levels, hyperglycemia and hypertension were defined according to the criteria of the AHA/NHLBI (2005)[12].

All analyses were performed with SAS version 9.1 (SAS Institute, INC., Cary, North Carolina). Distributions of genotypes were tested for deviation from HWE by chi-square analyses (PROC ALLELE). Associations with individual MetS features and co-occurrence of MetS features were tested. To optimize precision, subjects who changed phenotype between the two rounds were excluded. This means that subjects being e.g. hypertensive in one round and normotensive in the other round or vice versa were excluded. All analyses were adjusted for age and sex.

In a first series of analyses, the association between individual MetS features and each SNP was analyzed by log-linear models. The prevalence ratios of change per allele were calculated with an additive genetic model. To avoid chance findings we only followed up those SNPs which were related to multiple MetS features with P ≤ 0.01. We determined the expected number of SNPs related to 2 or more MetS features with P < 0.01 by chance alone and under the assumption of independent random outcomes using the following formula: Chance (P ≤ 0.01 for SNP_1 - MetS feature_1 association) * Chance (P ≤ 0.01 for the SNP_2 - MetS feature_2 association) * Chance (association 1 and 2 in the same direction) * number of MetS feature combinations * number of SNPs. The expected number appeared to be 0.12 (0.01 * 0.01 * 0.5 * 6 * 373). Subsequently we tested whether the number of observed SNPs associated with 2 or more MetS features differed significantly from the expected 0.12 SNPs.

In our study abdominal obesity and decreased HDL-cholesterol appeared to be the MetS features both associated with the same SNPs. In a second series of analyses, it was tested if the association between these SNPs and HDL-cholesterol was independent of the association with abdominal obesity, and vice versa. This was done both by adjustment and by stratification. The HDL-cholesterol analyses were adjusted for abdominal obesity and vice versa. For stratified analyses, the association with abdominal obesity was analyzed in subjects with high HDL-cholesterol levels. Low HDL-cholesterol was analyzed in subjects without abdominal obesity.

## Results

Baseline characteristics among the 3575 subjects of the Doetinchem cohort are presented in table 1. Hypertension was the most prevalent MetS feature (41.6% of the subjects were stable hypertensive and 32.3% of the subjects were stable normotensive). The least prevalent MetS feature was low HDL-cholesterol (18.7% were stable for low HDL and 64.1% were stable for high HDL). The most frequent combination of co-occurring MetS features was hypertension and abdominal obesity (14.5% were stable positive, 21.2% were stable negative). The least frequent combination was decreased HDL-cholesterol levels and hyperglycemia (3.5% were stable positive, 32.6% were stable negative).

**Table 1:** Characteristics of 3575 subjects of the Doetinchem Cohort in round 2 and 3

	Round 2: 1993-1997	Round 3: 1998-2002
Age (yr)	46.5 (9.7)	51.5 (9.7)
Sex (% men)	47.8	47.8
Waist circumference (cm)	90.2 (11.1)	92.9 (11.4)
Increased waist circumference (%)^a^	31.3	40.3
Glucose levels (mmol/L)^b^	5.3 (1.3)	5.4 (1.5)
Diabetic medication (%)	0.8	2.3
Hyperglycemia (%)^a^	28.8	33.6
HDL-cholesterol (mmol/L)	1.38 (0.38)	1.37 (0.39)
Low HDL-cholesterol (%)^a^	25.4	29.0
Diastolic Blood Pressure (mm Hg)	79.9 (10.6)	81.4 (10.7)
Systolic Blood Pressure (mm Hg)	125.1 (16.4)	129.3 (18.01)
Hypertension (%)^a^	50.8	58.5
Blood Pressure lowering medication (%)	6.5	11.0
MetS-score (number of features)	1.34 (1.1)	1.61 (1.1)
Metabolic syndrome prevalence (%)	14.9	22.7

Data are presented as means (standard deviation) or %

^a^Abdominal obesity, hyperglycemia, low HDL, hypertension and MetS are defined according to the criteria of AHA-NHLBI (2005). Abdominal obesity: ♂ ≥ 102 cm; ♀ ≥ 88 cm; Low HDL: ♂ < 1.0; ♀ < 1.3 mmol/L; hypertension: ≥ 130/85 mm Hg or hypertensive medicine; Hyperglycemia ≥ 5.6 (mmol/L) or glucose lowering medication; MetS is defined as having 3 MetS features measured in Doetinchem Cohort

^b^Non-fasting values

19 SNPs were related to at least one of the stable MetS features with P < 0.01 (table 2). Two of them, *Ile405Val (rs5882) *in the *Cholesteryl Ester Transfer Protein (CETP) *gene and *Cys112Arg (rs429358) *in the *Apolipoprotein E (APOE) *gene were related to 2 MetS features each with P < 0.01. This number differs significantly from the expected 0.12 SNPs to be associated with two features or more by chance alone (p < 0.005 chi-square with Yates correction). Both SNPs were in HWE (*Ile405Val *P = 0.21; *Cys112Arg *P = 0.48). The minor *Val *allele of *Ile405Val *in the *CETP *gene was associated with both a decreased prevalence of low HDL-cholesterol levels (PR/allele 0.76, 95%CI 0.69; 0.86) and a decreased prevalence of abdominal obesity (PR/allele 0.90, 95%CI 0.83; 0.97) (table 3). The minor *Arg *allele of the *Cys112Arg *in the *APOE *gene was associated with an increased prevalence of low HDL-cholesterol levels (PR/allele 1.21, 95% CI 1.07; 1.37) and an increased prevalence of abdominal obesity (PR/allele 1.12, 95% CI 1.03; 1.23) (table 4). Results for both SNPs remained significant after adjusting the abdominal obesity analyses for HDL-cholesterol and vice versa. Further analyses showed that both SNPs were associated with the simultaneous occurrence of abdominal obesity and low HDL-cholesterol levels, with decreased HDL-cholesterol levels in a subgroup of people without abdominal obesity, and with abdominal obesity in a subgroup of people with normal HDL-cholesterol levels (table 3; table 4).

**Table 2:** SNP's associated (P < 0.01) with stable MetS features among subjects of the Doetinchem Cohort over 2 surveys (1993-1997; 1998-2002)

SNP	MAF	Gene	PR/allele (95%CI)	P-Value
*Hyperglycemia (n = 2280)*				
rs1137101	0.46	LEPR	0.84 (0.76; 0.93)	0.001
rs3842748	0.21	INS-IGF2	1.20 (1.07; 1.35)	0.002
rs6795441	0.45	RAF1	0.86 (0.77; 0.95)	0.003
rs7903146	0.29	TCF7L2	1.17 (1.05; 1.30)	0.005
rs1143634	0.24	IL1B	1.17 (1.05; 1.31)	0.005
*Abdominal obesity (n = 2931)*				
rs35724	0.38	NR1H4	0.91 (0.85; 0.97)	0.005
rs10860603	0.14	NR1H4	0.86 (0.78; 0.96)	0.006
rs1800796	0.04	IL6	0.77 (0.64;0.93)	0.007
rs5882	0.31	CETP	0.90 (0.83;0.97)	0.007
rs429358	0.16	APOE	1.12 (1.03;1.23)	0.007
*Hypertension (n = 2643)*				
rs130005	0.10	CREBBP	0.89 (0.82; 0.97)	0.006
rs3759324	0.25	SCCN1A	1.07 (1.02; 1.12)	0.009
*Low HDL-Cholesterol (n = 2959)*				
rs1800777	0.03	CETP	1.60 (1.56;2.32)	3.3 E-12
rs3208305	0.30	LPL	0.70 (0.63;0.79)	9.3 E-10
rs328	0.11	LPL	0.60 (0.49; 0.72)	1.2 E-7
rs5882	0.31	CETP	0.76 (0.69;0.86)	2.1 E-6
rs429358	0.16	APOE	1.21 (1.07;1.37)	0.001
rs174546	0.33	FADS1	1.18 (1.07;1.30)	0.001
rs780094	0.36	GCKR	1.17 (1.06; 1.29)	0.002
rs268	0.02	LPL	1.45 (1.12; 1.86)	0.004
rs5275	0.31	PTGS2	1.15 (1.04; 1.27)	0.006

MAF = Minor allele frequency; PR = Prevalence Ratio; ^b ^Prevalence ratios are expressed per minor allele assuming an additive genetic model

**Table 3:** Association of *Ile405Val (rs5882) *in the *CETP *gene with abdominal obesity and low HDL-cholesterol levels

**Outcome***^a ^*	Ile/Ile	Ile/Val	Val/Val	**PR/allele (95%CI)**^b^	P-trend
**Prevalence of low HDL**^c^					
Overall *(n = 669, total n = 2959)*	26.0%	20.6%	14.4%	0.76 (0.69;0.86)	< .0001
Adjusted for abdominal obesity *(n = 669, total n = 2959)*	24.7%	21.3%	16.0%	0.83 (0.74;0.93)	0.002
Among subjects without abdominal obesity *(n = 252, total n = 1684)*	18.4%	13.0%	7.8%	0.68 (0.56;0.82)	< 0.001
**Prevalence of abdominal obesity**^d^					
Overall *(n = 958, total n = 2931)*	34.7%	31.9%	26.4%	0.90 (0.83;0.97)	0.0072
Adjusted for low HDL *(n = 958, total n = 2931)*	33.1%	30.4%	27.0%	0.92 (0.83;1)	0.05
Among subjects with high HDL levels *(n = 470, total n = 1902)*	27.9%	22.8%	18.7%	0.82 (0.73;0.93)	0.0014
**Prevalence of both low HDL and abdominal obesity**^e ^					
Overall *(n = 298, total n = 1730)*	19.5%	16.3%	11.2%	0.81 (0.69;0.94)	0.0076

PR = Prevalence Ratio

^a ^All analyses are adjusted for age and sex

^b ^Prevalence ratios are expressed per minor VAL allele assuming an additive genetic model

^c ^Subjects with low HDL-cholesterol in round 2, but not in round 3 or vice versa, were excluded

^d ^Subjects with abdominal obesity in round 2, but not round 3 or vice versa, were excluded

^e ^Only subjects with either both abdominal obesity and low HDL-cholesterol levels in round 2 and 3 or with both no abdominal obesity and high HDL-cholesterol levels were included. Subjects without abdominal obesity and with high HDL-cholesterol levels in round 2 and 3 were used as the reference category

**Table 4:** Association of *Cys112Arg (rs429358) *in the *APOE *gene with abdominal obesity and low HDL-cholesterol levels

**Outcome***^a ^*	Cys/Cys	Cys/Arg	Arg/Arg	**PR/allele (95%CI) **^**b**^	P-trend
**Prevalence of low HDL**^c^					
Overall *(n = 669, total n = 2959)*	21.2%	25.5%	32.3%	1.21 (1.07;1.37)	0.0013
Adjusted for abdominal obesity *(n = 669, total n = 2959)*	21.0%	25.5%	30.2%	1.20 (1.06;1.36)	0.005
Among subjects without abdominal obesity *(n = 252, total n = 1684)*	13.5%	18.0%	26.0%	1.35 (1.11; 1.65)	0.0031
**Prevalence of abdominal obesity**^d^					
Overall *(n = 958, total n = 2931)*	31.2%	35.8%	39.6%	1.12 (1.03;1.23)	0.0074
Adjusted for low HDL *(n = 958, total n = 2931)*	30.1%	33.1%	38.9%	1.12 (1.0l;1.25)	0.04
Among subjects with high HDL levels *(n = 470, total n = 1902)*	23.5%	27.3%	34.5%	1.16 (1.01;1.33)	0.03
**Prevalence of both low HDL and abdominal obesity**^e^					
Overall *(n = 298, total n = 1730)*	15.7%	20.7%	27.8%	1.29 (1.08;1.54)	0.0045

PR = Prevalence Ratio

^a^All analyses are adjusted for age and sex

^b ^Prevalence ratios are expressed per minor ARG allele assuming an additive genetic model

^c ^Subjects with low HDL-cholesterol in round 2, but not in round 3 or vice versa, were excluded

^d ^Subjects with abdominal obesity in round 2, but not round 3 or vice versa, were excluded

^e ^Only subjects with either both abdominal obesity and low HDL-cholesterol levels in round 2 and 3 or with both no abdominal obesity and high HDL-cholesterol levels were included. Subjects without abdominal obesity and with high HDL-cholesterol levels in round 2 and 3 were used as the reference category

The *Cys112Arg *genotype of the *APOE *gene is part of the ε2, ε3, ε4 haplotype. Results of the ε2ε3ε4 haplotype analyses were similar to the results of the *Cys112Arg *analyses. Compared to the ε3/ε3 isoform, the ε4/- isoforms (ε3/ε4 and ε4/ε4) were associated with an increased prevalence of low HDL-cholesterol levels (PR 1.24, 95%CI 1.07; 1.44) and an increased prevalence of abdominal obesity (PR 1.13, 95%CI 1.01; 1.26). No associations were found with the ε2/- isoforms (ε3/ε2 and ε2/ε2).

## Discussion

In this explorative study of 373 SNPs, mainly located in pathways related to lipid and glucose metabolism, we found a significant association between the *Ile405Val *genotype in the *CETP *gene and the *Cys112Arg *genotype in the *APOE *gene, with multiple features of the metabolic syndrome, i.e. the prevalence of abdominal obesity and prevalence of low HDL-cholesterol. For both SNPs, the association with abdominal obesity was partly independent of the association with HDL-cholesterol, and vice versa. No, association was found between SNPs in genes involved in glucose metabolism or blood pressure regulation and multiple MetS features.

In humans, CETP and ApoE are expressed in the liver and in peripheral tissues, such as adipose tissue [13,14]. Both genes are involved in plasma lipid homeostasis. CETP stimulates the clearance of HDL-cholesterol from plasma [14]. Furthermore, CETP increases the formation of small dense LDL particles and triglycerides [15]. ApoE removes atherogenic lipoproteins, such as VLDL, from the circulation [16]. This results in lower cholesterol and triglyceride levels. Besides having a role in lipid homeostasis, a few studies indicate that CETP and ApoE may be involved in other metabolic processes such as weight regulation. For example, APOE plays a role in the deposition of dietary fat in adipose tissue [17]. As CETP is synthesized in the adipose tissue, CETP may affect adipose tissue characteristics [18].

The *Ile405Val *polymorphism in the *CETP *gene induces a change in amino acid sequence. Therefore it is likely to be a functional SNP. In our study, the Val allele of the *Ile405Val *genotype was associated with a lower prevalence of abdominal obesity and a lower prevalence of low HDL-cholesterol levels. The stratified and adjusted analyses in our study suggested that the association with prevalence of abdominal obesity and prevalence of low HDL-cholesterol levels was partly independent of each other. This suggests that CETP regulates weight and HDL-cholesterol via independent pathways.

In line with our results, a meta-analysis of 29 studies, showed that *Val *allele carriers had higher HDL levels [19]. Furthermore, a Chinese case-control study in 934 obesity cases and 924 controls showed a decreased obesity risk for *Val/Val *homozygotes, which persisted after adjustment for HDL-cholesterol levels [20]. In previous studies, the *405Val *allele has been associated with lower CETP mass and lower CETP activity [19]. Lower CETP plasma levels are correlated with a lower obesity risk [21]. The *405Val *allele has also been associated with other positive health outcomes such as, increased HDL and LDL particle size [15], decreased coronary heart disease risk [22], and increased longevity [15], all of which are related to the MetS. In summary, cumulative evidence indicates that *Ile405Val *is involved in several metabolic processes, including lipid level control and weight regulation.

The *Cys112Arg *genotype of the *APOE *gene is a non-synonymous genotype. Together with *Arg158Cys (rs7412)*, the *Cys112Arg *forms the ε2ε3ε4 haplotype. The ε2, ε3 and ε4 ApoE isoforms differ markedly on the structural and functional level [16]. In our study the *Arg *allele of the *Cys112Arg *genotype was associated with an increased prevalence of low HDL-cholesterol levels and an increased prevalence of abdominal obesity. Again the stratified and adjusted analyses suggested that the associations with the prevalence of abdominal obesity and prevalence of low HDL-cholesterol levels were partly independent of each other. The ε4 isoform showed a similar, though less pronounced, pattern of associations. No associations were observed with *Arg158Cys *or ε2 isoform, of the ε2ε3ε4 haplotype.

Previous studies generally focused on the ε2, ε3 and ε4 haplotype and did not take associations with the individual *Arg158Cys *and *Cys112Arg *into account. In line with our study, the ε4 isoform was associated with a more detrimental metabolic profile in most studies. A meta-analysis of 19 studies in 9751 subjects, showed that ε3/ε4 carriers had lower HDL-cholesterol levels than ε3/ε3 carriers [23]. Most studies showed either a positive [24-27] or no [28-31] association between the ε4 isoform and body weight. However, some showed a negative association [30,31]. Arbones Mainar et al. [28] showed that compared to ApoE3 mice, ApoE4 mice fed a western diet were more prone to the development of several MetS features, such as increased insulin resistance, decreased fat tolerance and increased fat cell size. However, they gained less body weight. This suggests that the positive association between the ε4 isoform and abdominal obesity may be driven by the development of other MetS features, such as insulin resistance [28]. Furthermore, these results suggest that the ε4 isoform may be associated with MetS. This has indeed been shown in other epidemiological studies [25,26,29].

Strength of our pathway driven candidate gene study was the relatively large sample size. Contrast and precision were increased by exclusively including people with consistent MetS phenotype, i.e. classified as healthy or not healthy for a particular metabolic phenotype over two measurement rounds. Furthermore we tried to keep the probability of chance findings low by including only those SNPS that were related to two or more MetS features with P < 0.01 into the second round of data-analysis. We found 2 SNPs, which differed significantly from the expected 0.12 SNPs (p < 0.005 chi-square with Yates correction). However, the 0.12 expected SNPs were obtained assuming independent random outcomes. As HDL-cholesterol and abdominal obesity are not completely independent, this assumption is partly violated. However, the associations with abdominal obesity and HDL-cholesterol remained significant in our stratified and adjusted analyses. A weakness of our study may be that blood samples were taken from non-fasting subjects. This may have randomly affected the glucose measurements. Another weakness is that triglycerides levels were not measured in our study. Therefore, we may have missed SNPs which were related to hypertriglyceridemia and one or two other MetS feature. For example, the *CETP Ile405Val *mutation has been associated with triglycerides in previous studies [19]. We therefore expect that in our study population this SNP will not only be associated with HDL-cholesterol and abdominal obesity, but also with triglyceride levels.

In this explorative study of 373 SNPs among 3575 subjects, we emphasized on the intricate links between several MetS features. We have showed that two SNPs, mainly known for their role in lipid metabolism, influenced both abdominal obesity and low HDL-cholesterol levels, partly independent of each phenotype. If the pleiotropic effects of these genes are further confirmed by others it might be possible to develop medication which increases HDL-cholesterol leves and reduces waist circumference, and so affects the development of MetS

## Competing interests

The authors declare that they have no competing interests.

## Authors' contributions

METD, JMAB, EJMF and SI participated in performing the research. CMP, JMAB and EJMF analyzed the data. All authors participated in interpretation of the data. CMP drafted the manuscript. All authors read and approved the final manuscript.
